# Excessive Daytime Sleepiness in Tension-Type Headache: A Population Study

**DOI:** 10.3389/fneur.2019.01282

**Published:** 2019-12-03

**Authors:** Kyung Min Kim, Jiyoung Kim, Soo-Jin Cho, Won-Joo Kim, Kwang Ik Yang, Chang-Ho Yun, Min Kyung Chu

**Affiliations:** ^1^Department of Neurology, Severance Hospital, Yonsei University College of Medicine, Seoul, South Korea; ^2^Department of Neurology, Bio Medical Research Institute, Pusan National University Hospital, Pusan National University School of Medicine, Busan, South Korea; ^3^Department of Neurology, Dongtan Sacred Heart Hospital, Hallym University College of Medicine, Hwaseong, South Korea; ^4^Department of Neurology, Gangnam Severance Hospital, Yonsei University College of Medicine, Seoul, South Korea; ^5^Sleep Disorders Center, Department of Neurology, Soonchunhyang University College of Medicine, Cheonan Hospital, Cheonan, South Korea; ^6^Department of Neurology, Bundang Clinical Neuroscience Institute, Seoul National University Bundang Hospital, Seongnam, South Korea

**Keywords:** excessive daytime sleepiness, tension-type headache, headache, sleep, epidemiology

## Abstract

Excessive daytime sleepiness (EDS) is a prevalent sleep-related complaint across the general population and has been reported to be associated with headache. Tension-type headache (TTH) is the most commonly encountered headache and accounts for a significant amount of disease burden. However, the association between EDS and TTH is currently scarce. In the present study, we investigated the impact of EDS on the prevalence and clinical presentation of TTH. We utilized data from the Korean Headache-Sleep Study, a national survey that sought to identify headache and sleep characteristics in Korean adults. Participants with an Epworth sleepiness scale score greater or equal to 11 were considered as having EDS. Of the 2,695 participants enrolled, 570 (21.2%) and 313 (11.6%) had TTH and EDS, respectively. EDS was highly prevalent in individuals with chronic tension-type headache (CTTH) than in those without headache (35.7 vs. 9.4%, *p* < 0.001). The prevalence of EDS in episodic tension-type headache (ETTH) individuals with a headache frequency <1 per month (8.3%, *p* = 0.511) and ETTH individuals with a headache frequency of 1–14 per month (13.5%, *p* = 0.054) was not significantly different from that in individuals without headache (9.4%). TTH participants with EDS had a higher headache frequency per month (4.3 ± 8.1 vs. 1.7 ± 4.2, *p* = 0.013), more severe headache intensity (Visual Analog Scale, 5.0 [3.0–6.0] vs. 4.0 [3.0–6.0], *p* = 0.008), a higher impact of headache (Headache Impact Test-6 score, 47.1 ± 7.3 vs. 43.5 ± 7.6, *p* < 0.001), and a higher prevalence of depression (12.7 vs. 3.2%, *p* < 0.001) than TTH participants without EDS. Consequently, CTTH is associated with higher EDS prevalence compared to ETTH and without headache. Moreover, TTH with EDS had more severe TTH symptoms compared to TTH without EDS.

## Introduction

Excessive daytime sleepiness (EDS) is a well-known complaint encountered in sleep clinics and can occur in up to 12% of the general population (1, 2). EDS can result in reduced quality of life, accidents, reduced performance, and cognitive and mood impairment (3–5). Various neurological and psychological conditions have been reported to contribute to EDS. Patients with common neurological disorders such as parkinsonism, multiple sclerosis, stroke, and epilepsy often present with EDS. Moreover, a previous study showed that individuals with depression, anxiety, and schizophrenia often reported EDS (5).

Tension-type headache (TTH) is the most prevalent type among primary headaches and individuals with TTH usually have mild or no disability. However, some individuals with TTH experience disabling symptoms that result in a significant amount of disability and reduced quality of life (6). Because of its high prevalence, the disease burden of TTH is estimated to be greater than that of migraine (7).

Previous studies have reported a significant association between EDS and headache. It has been reported that migraineurs experience more EDS than non-migraineurs, with EDS being associated with the exacerbation of migraine symptoms (8, 9). Frequency of EDS was shown to be higher among individuals with episodic and chronic migraine than among individuals without headache (10, 11). However, there is currently limited information on the prevalence of EDS in TTH individuals and the impact of EDS on TTH. Therefore, we conducted a study with a population-based sample to investigate the following: (1) the prevalence of EDS in TTH individuals; (2) the clinical presentations of TTH associated with EDS; (3) The factors contributing EDS occurrence in TTH individuals.

## Materials and Methods

### Study Protocol

We conducted a cross-sectional study to analyze data from the Korean Headache-Sleep Study (KHSS), which is a national population study for headache and sleep. Detailed methods of this survey were previously described (12). Briefly, two-stage random cluster sampling was used to reflect the regional distribution in Korea except for Jeju-do. Well-trained interviewers performed this survey by door-to-door visiting using a structured questionnaire. The questionnaire was composed of questions asking yes-no answers or Likert scales. Each participant provided informed consent before the survey and the study protocol was reviewed and approved by the Institutional Review Board and Ethics Committee of Hallym University Sacred Heart Hospital (Approval No. 2011-I077).

### Headache Assessment

Participants that experienced more than 1 min of headache during the previous year, they were classified as having headache. If participants did not experience a headache lasting more than 1 min during the previous year, they were classified as participants without headache.

### TTH Assessment

For TTH diagnosis, we used diagnostic criteria B to D for infrequent episodic TTH (code 2.1) in the third edition, beta version of the International Classification of Headache Disorders (ICHD-3 beta) (B: lasting from 30 min to 7 days; C: at least two of the following four characteristics [i.e., bilateral location, pressing or tightening (non-pulsating) quality, mild or moderate intensity, and not aggravated by routine physical activity such as walking or climbing stairs]; D: attacks associated with both of the following: no nausea or vomiting and no more than one of photophobia or phonophobia). Participants who fulfilled these criteria were classified as having TTH. Diagnostic criterion A (at least 10 episodes of headache) was not applied in TTH diagnosis. Thus, TTH individuals included in this study had infrequent TTH (code 2.1), frequent TTH (code 2.2), and chronic TTH (code 2.3). If a participant classified as having headache and fulfilling B to D criteria for infrequent episodic TTH, she/he was classified as having TTH. Based on ICHD-3 beta, participants that fulfilled with both diagnostic criteria for probable migraine (PM) and TTH were considered as having TTH.

### EDS Assessment

The Epworth Sleepiness Scale (ESS) was used for EDS assessment. The total ESS score ranges from 0 to 24 points. Participants with a score equal to or >11 were considered as having EDS. We used a previously verified Korean version of ESS (13).

### Short Sleep Duration and Poor Sleep Quality Assessment

Short sleep duration was defined as an average sleep duration of 6 h or less. The average sleep duration was calculated using the following formula; [(workday sleep duration × 5) + (free day sleep duration × 2)]/7; the sleep durations on the weekdays and free days were independently measured using the survey data on sleep onset and wakeup time.

The Pittsburgh Sleep Quality Index (PSQI) was adopted to assess sleep quality. The PSQI consists of 19 questions on various aspects of perceived sleep status. The total PSQI score ranges from 0 to 21 points. Poor sleep quality was defined as a PSQI score of 6 or more (14).

### Anxiety and Depression Assessment

Anxiety was assessed using the Goldberg Anxiety Scale (GAS), which is composed of a total of nine items, four screening items, and five supplementary items (15). Participants who had five or more positive answers for all the items, with two or more being for the screening items, were considered as having anxiety. The Korean version GAS has a sensitivity of 82.0% and a specificity of 94.4% for diagnosing anxiety (16). This study evaluated depression using the Patient Health Questionnaire-9 (PHQ-9) (17). The PHQ-9 was developed based on the DSM-IV criteria for major depression and has been widely used in epidemiological studies (18–20). Response options were rated on a four -point scale from 0 to 3, regarding the past 2 weeks experienced. Participants with a PHQ-9 score of 10 or more were classified as having depression. The sensitivity and specificity of the Korean version of PHQ-9 were previously validated as 81.1 and 89.9%, respectively (21).

### Statistical Analysis

Normality of distribution was evaluated using Kolmogorov–Smirnov test. After normality of distribution was confirmed, Student's *t*-test was used to compare two groups. If the normality of distribution was not confirmed, Mann–Whitney *U*-test was used to compare two groups. Chi-square test was used to compare the prevalence rates. Univariable and multivariable analyses were performed to determine factors contributing to EDS among TTH individuals. For univariable analyses, factors with significant differences between TTH individuals with EDS and those without EDS were considered. For multivariable analyses, three models were developed to examine the association between EDS and TTH. In Model 1, sociodemographic factors (sex, age, size of residential area, and level of education) were adjusted. In Model 2, sociodemographic factors and sleep-related parameters (short sleep duration and poor sleep quality) were adjusted. In Model 3, sociodemographic factors and psychiatric comorbidity (anxiety and depression) were adjusted. In the final model (Model 4), sociodemographic factors, sleep-related parameters, and psychiatric comorbidity were adjusted. Statistical Package for Social Sciences version 22.0 (SPSS 22.0; IBM, Armonk, NY, USA) was used for all statistical analysis. Statistical significance was defined as a two-tailed *p* < 0.05.

Several variables had missing data because of non-response. Available data were used for the final analysis. There was no missing data for all 2,695 participants except for educational level, which had 26 non-respondents.

## Results

### Survey

**Figure 1:**
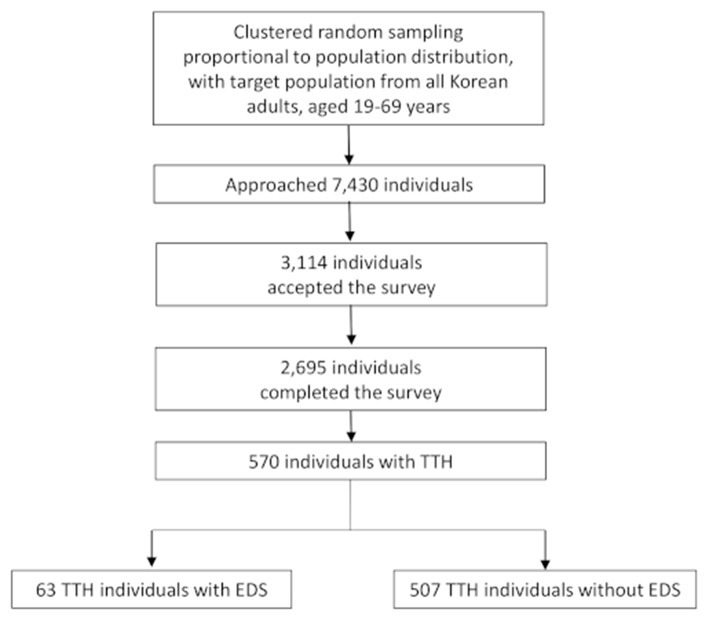
Flow chart depicting the participation of subjects in the Korean Headache-Sleep Study.

We approached 7,430 subjects and 3,114 (41.9%) subjects agreed to respond to the survey. Finally, 2,695 (36.3%) participants completed the survey (Figure 1). Collected data on demographic factors (age, sex, size of residential area, and level of education) revealed no significant difference from that of the Korean general population (Table 1).

**Table 1:** Demographic characteristics of the survey participants; total population; and participants with tension-type headache (TTH) and excessive daytime sleepiness (EDS).

	**Survey participants, N (%)**	**Total population, N (%)**	***P***	**TTH, N, % (95% CI)**	**EDS, N, % (95% CI)**
**Sex**					
Men	1,345 (49.3)	17,584,365 (50.6)	0.854^*^	268, 19.9 (17.8–22.0)	161, 12.0 (10.2–13.7)
Women	1,350 (50.7)	17,198,350 (49.4)		302, 22.3 (20.1–24.6)	152, 11.3 (9.6–12.9)
**Age**					
19–29	542 (20.5)	7,717,947 (22.2)	0.917^*^	119, 22.0 (18.5–25.5)	52, 9.6 (7.1–12.1)
30–39	604 (21.9)	8,349,487 (24.0)		127, 21.0(17.8–24.3)	71, 11.8 (9.2–14.3)
40–49	611 (23.1)	8,613,110 (24.8)		131, 21.4 (18.2–24.7)	62, 10.1 (7.7–12.5)
50–59	529 (18.9)	6,167,505 (17.7)		107, 20.2 (16.8–23.7)	66, 12.5 (9.7–15.3)
60–69	409 (15.6)	3,934,666 (11.3)		86, 21.0 (17.1–25.0)	62, 15.2 (11.7–18.6)
**Size of residential area**					
Large city	1,248 (46.3)	16,776,771 (48.2)	0.921^*^	251, 20.1 (17.9–22.4)	150, 12.0 (10.2–13.8)
Medium-to-small city	1,186 (44.0)	15,164,345 (43.6)		243, 20.5 (18.2–22.8)	134, 11.3 (9.5–13.1)
Rural area	261 (9.7)	2,841,599 (8.2)		76, 29.1 (23.6–34.7)	29, 11.1 (7.3–14.9)
**Education level**					
Middle school or less	393 (14.9)	6,608,716 (19.0)	0.752^*^	96, 24.5 (20.1–28.7)	58, 14.8 (11.2–18.3)
High school	1,208 (44.6)	15,234,829 (43.8)		247, 20.5 (18.2–22.7)	135, 11.2 (9.4–13.0)
College or more	1,068 (39.6)	12,939,170 (37.2)		223, 20.9 (18.4–23.3)	119, 11.1 (9.3–13.0)
Not responded				4, 15.4 (0.5–30.2)	1, 3.8 (0.0–11.8)
Total	2,695 (100.0)	34,782,715 (100.0)		570, 21.2 (19.6–22.7)	313, 11.6 (10.4–12.8)

* *Comparison of sex, age group, size of residential area, and educational level distributions between the sample in the present study and the total population of Korea*.

*TTH, tension-type headache; EDS, excessive daytime sleepiness; N, number; CI, confidence interval*.

### Prevalence of TTH, Non-headache, and EDS

Of the 2,695 participants, 570 (21.2%) had TTH while 1,422 (52.8%) did not have a headache during the previous year. Among TTH individuals, 113 (19.8%) also had PM. Three-hundred and thirteen (11.6%) participants were classified as having EDS (Table 1).

### Prevalence of Anxiety, Depression, Short Sleep Duration, and Poor Sleep Quality

A total of 268 (9.9%) participants had anxiety while 116 (4.3%) had depression. The prevalence rates of anxiety (9.5 vs. 5.3%, *p* = 0.001) and depression (4.2 vs. 1.8%, *p* = 0.001) were significantly higher among TTH individuals than among individuals without headache. A total of 469 (17.4%) participants reported having an average sleep duration of <6 h, and were classified as having short sleep duration. A total of 715 (26.5%) participants were classified as having poor sleep quality.

### EDS Prevalence in Individuals With TTH

Among the 570 participants with TTH, 63 (11.1%) reported experiencing EDS. The EDS prevalence in individuals with TTH was not significantly different from that in individuals without headache (11.1 vs. 9.4%, *p* = 0.271). The prevalence of EDS was not significantly different between TTH participants fulfilling PM criteria and TTH participants not-fulfilling PM (11.6 vs. 8.8%, *p* = 0.404).

**Figure 2:**
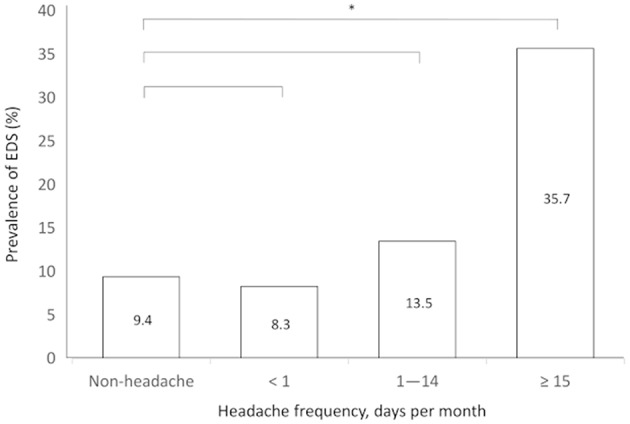
Prevalence of excessive daytime sleepiness according to the frequency of tension-type headache. ^*^p < 0.05.

EDS prevalence was analyzed with regard to headache frequency. Individuals with 15 or more TTH attacks per month had a significantly higher EDS prevalence compared to that those without headache (35.7 vs. 9.4%, *p* < 0.001). Individuals with 1–14 TTH attacks per month (13.5 vs. 9.4%, *p* = 0.054) and with <1 attack per month (8.3 vs. 9.4%, *p* = 0.511) did not have a significantly different EDS prevalence compared to those without headache (Figure 2).

### Clinical Presentation of TTH According to the Presence of EDS

Osmophobia and depression were more prevalent among TTH participants with EDS compared to those without. TTH participants with EDS showed significantly higher headache frequency, visual analog scale (VAS) scores for headache intensity, and Headache Impact Test-6 scores compared to those without EDS (Table 2).

**Table 2:** Demographics and clinical presentation of individuals with tension-type headache (TTH) according to the presence of excessive daytime sleepiness (EDS).

	**TTH with EDS (*N* = 63)**	**TTH without EDS (*N* = 507)**	***P***
**Demographics**			
Mean age ± SD (years)	45.4 ± 13.5	42.4 ± 13.7	0.092
Women, *N* (%)	31 (49.2)	271 (53.5)	0.524
**Headache characteristics**			
Bilateral pain, *N* (%)	36 (57.1)	338 (66.7)	0.133
Non-pulsating quality, *N* (%)	24 (38.1)	203 (40.0)	0.766
Mild-to-moderate severity, *N* (%)	62 (98.4)	498 (98.2)	0.915
Non-aggravation by movement, *N* (%)	51 (81.0)	400 (78.9)	0.705
**Associated symptoms**			
Photophobia, *N* (%)	6 (9.5)	40 (7.9)	0.653
Phonophobia, *N* (%)	22 (34.9)	159 (31.4)	0.567
Osmophobia, *N* (%)	17 (27.0)	78 (15.4)	0.020
Headache frequency per month, mean ± SD	4.3 ± 8.1	1.7 ± 4.2	0.013
VAS score, mean ± SD	4.9 ± 1.6	4.3 ± 1.9	0.007
HIT-6 score, mean ± SD	47.1 ± 7.3	43.5 ± 7.6	<0.001
Anxiety	9 (14.3)	45 (8.9)	0.167
Depression	8 (12.7)	16 (3.2)	<0.001

*TTH, tension-type headache; EDS, excessive daytime sleepiness; N, number; SD, standard deviation; VAS, visual analog scale; HIT-6, headache impact test-6*.

### Univariable and Multivariable Analysis for Contributing Factors to EDS Among TTH Individuals

Univariable analysis showed that depression (odds ratio [OR] = 4.5, 95% confidence interval [CI] = 1.8–10.9) and poor sleep quality (OR = 5.0, 95% CI = 2.9–8.7) were associated with an increased risk of EDS in TTH individuals. Multivariable analysis of sociodemographic factors revealed no statistically significant association with EDS (Model 1). In Model 2, which included sociodemographic factors, depression, and anxiety, depression (OR = 4.5, 95% CI = 1.7–11.7) showed increased ORs for EDS. In Model 3, which included sociodemographic factors, short sleep time, and poor sleep quality, poor sleep quality (OR = 6.6, 95% CI = 3.6–12.1) showed increased ORs for EDS; however, high school graduates (OR = 0.4, 95% CI = 0.1–0.9) showed decreased ORs for EDS. In Model 4, which included sociodemographic factors, anxiety, depression, short sleep time, and poor sleep quality, poor sleep quality (OR = 6.0, 95% CI = 3.2–11.2) showed increased ORs for EDS while high school graduates (OR = 0.4, 95% CI = 0.1–0.9) showed decreased ORs for EDS (Table 3).

**Table 3:** Logistic regression for the contributing factors of excessive daytime sleepiness (EDS) among individuals with tension-type headache (TTH).

	**Univariable analysis OR, 95% CI**	**Multivariable analysis OR, 95% CI (Model 1)**	**Multivariable analysis OR, 95% CI (Model 2)**	**Multivariable analysis OR, 95% CI (Model 3)**	**Multivariable analysis OR, 95% CI (Model 4)**
Sex	1.2 (0.7–2.0)	1.2 (0.7–2.1)	1.2 (0.7–2.1)	1.2 (0.7–2.2)	1.2 (0.7–2.2)
**Age**					
19–29	Reference	Reference	Reference	Reference	Reference
30–39	1.5 (0.6–3.6)	1.6 (0.7–4.0)	1.5 (0.6–3.8)	1.4 (0.6–3.6)	1.4 (0.6–3.5)
40–49	1.7 (0.7–4.0)	1.9 (0.8–4.6)	1.9 (0.8–4.6)	2.0 (0.8–5.0)	2.0 (0.8–5.0)
50–59	1.4 (0.6–3.5)	1.2 (0.4–3.5)	1.3 (0.4–3.6)	1.0 (0.3–2.9)	1.0 (0.3–3.1)
60–69	2.2 (0.9–5.4)	1.7 (0.6–5.3)	1.8 (0.6–5.4)	1.5 (0.5–4.6)	1.5 (0.5–4.7)
**Size of residential area**					
Large city	Reference	Reference	Reference	Reference	Reference
Medium-to-small city	1.2 (0.7–2.0)	1.2 (0.7–2.0)	0.5 (0.2–1.2)	1.2 (0.7–2.1)	1.1 (0.6–2.0)
Rural area	0.4 (0.2–1.3)	0.4 (0.7–2.1)	0.6 (0.2–1.6)	0.4 (0.1–1.2)	0.4 (0.1–1.2)
**Educational level**					
Middle school or less	Reference	Reference	Reference	Reference	Reference
High school	0.6 (0.3–1.2)	0.5 (0.2–1.2)	0.5 (0.2–1.2)	0.4 (0.1–0.9)	0.4 (0.1–0.9)
College or more	0.7 (0.3–1.3)	0.6 (0.2–1.6)	0.6 (0.2–1.6)	0.4 (0.2–1.2)	0.4 (0.2–1.3)
Anxiety	1.7 (0.8–3.7)		1.2 (0.5–2.8)		1.1 (0.5–2.7)
Depression	4.5 (1.8–10.9)		4.5 (1.7–11.7)		1.8 (0.7–5.3)
Short sleep duration (<6 h)	1.2 (0.6–2.2)			0.5 (0.3–1.1)	0.5 (0.3–1.1)
Poor sleep quality (PSQI >5)	5.0 (2.9–8.7)			6.6 (3.6–12.1)	6.0 (3.2–11.2)

*Model 1, adjusted for sociodemographic factors (age, sex, size of residential area, educational level); Model 2, adjusted for sociodemographic factors, anxiety, and depression; Model 3: adjusted for sociodemographic factors, short sleep duration, and poor sleep quality; Model 4, adjusted for sociodemographic factors, anxiety, depression, short sleep duration, and poor sleep quality. OR, odds ratio; CI, confidence interval; PSQI, pittsburgh sleep quality index*.

## Discussion

The key findings of this study include: (1) The overall prevalence of EDS in TTH participants was similar to that in participants without headache; (2) The prevalence of EDS among individuals with chronic tension-type headache (CTTH) was higher than that in individuals with episodic tension-type headache (ETTH) and without headache; and (3) TTH participants with EDS had a higher headache frequency, severe headache intensity, increased impact of headache, and a higher prevalence of depression compared to TTH participants without EDS.

There have been limited reports on the prevalence and impact of EDS in TTH. The prevalence of EDS in CTTH participants was found to be similar to that in participants with chronic migraine (CM). It has also been reported that individuals with a headache frequency ≥80 per 3 months experience EDS more frequently compared to those with a headache frequency <80 per 3 months (22). Moreover, a previous study reported that EDS prevalence in TTH individuals did not significantly differ from that in individuals without headache (9). However, the prevalence of EDS among individuals with CTTH in comparison with those with ETTH and without headache has not yet been reported in a population-based setting. The present study is the first to report a significantly higher prevalence of EDS among individuals with CTTH compared to that in individuals with ETTH and individuals without headache.

Epidemiological studies have reported the prevalence of EDS to range from 8 to 30%. A Singaporean study reported an EDS prevalence of 9.0% (23). A Korean population-based study using data from the Korean Genome Epidemiology Study found an EDS prevalence of 12.2% (24). An Australian study reported an EDS prevalence of 15.3% (25). The similarity in EDS prevalence between this study and previous studies suggests that the assessment of EDS in this study is reliable.

The present study found that the overall prevalence of EDS in TTH individuals was similar to that in individuals without headache; however, the EDS prevalence in CTTH individuals was higher than in ETTH and individuals without headache. These findings are similar to those of a Norwegian study that reported that the overall EDS prevalence in TTH individuals was similar to that in non-headache individuals (9). In this previous study, the prevalence of EDS in CTTH, which was reported to be higher in individuals without headache (8, 9), was similar to that in individuals with CM. The prevalence of EDS in CTTH individuals (35.7%) in the present study was somewhat higher than that in the Norwegian study (20.8%). One possible explanation for this discrepancy is the difference in ethnicity and sociodemographic distribution between the two studies. A possible reason for sociodemographic distribution difference could be that while our study used data from the KHSS, which sampled participants proportionally to the regional distribution in Korea, the Norwegian study used a community-based sample aged 30–44 from Akershus County, Norway. Therefore, this difference in sociodemographic distribution may account for the discrepancy in the prevalence of EDS in CTTH individuals between the two studies. Moreover, the cultural difference in ways of reporting sleepiness could be another source of discrepancy. A previous study reported large variations in the EDS prevalence across the world (26), with the difference in survey methods being another possible explanation for the discrepancy.

EDS is defined as the inability to stay awake and alert during major working episodes of the day, resulting in periods of irrepressible need for sleep or unintended lapses into drowsiness or sleep, based on the third edition of International Classification of Sleep Disorders (27). EDS is one of the most disabling conditions caused by poor nocturnal sleep and can lead to poorer occupational and social functioning, and is strongly related to an increased risk of vehicular and occupational accidents (28, 29). The present study found that a significant proportion of individuals with TTH had EDS. Considering the effect of EDS and daytime dysfunction, some of burden by TTH could be attributed to EDS. Therefore, proper diagnosis and treatment of EDS in TTH could be a useful way to reduce burden of TTH.

Univariable analysis in the present study showed that depression and poor sleep quality among TTH individuals were significant factors for EDS. After adjusting for potential covariates, multivariable analyses only showed high school educational level and poor sleep quality as significant factors for EDS. These findings are similar to those of other studies on the association between EDS and migraine. A Norwegian study revealed that a high headache frequency and depression were significant factors for EDS in migraineurs and TTH individuals (9). Our previous study also found that depression and poor sleep quality were significant factors for EDS in migraineurs (8). These findings suggest that EDS in CM and CTTH may be improved by improving depression and poor sleep quality. Depression has been shown to be successfully treated by pharmacological and non-pharmacological treatment (30). A previous study reported that poor sleep quality was closely associated with insomnia and that insomnia could be successfully managed by improving sleep hygiene, behavioral cognitive therapy, pharmacological treatment, etc. (31). Considering that EDS is associated with an exacerbation of TTH symptoms, improving EDS may be helpful in improving TTH symptoms.

This study has several limitations. First, this study assessed EDS using the ESS rather than objective measurement. ESS is a simple self-administered questionnaire, and is the most widely used tool for assessing subjective sleepiness. The ESS score is highly correlated with partners' independent reports of the subjects' report (32). There is evidence on the validity of ESS and its usefulness in assessing EDS and its changes after treatment (33–35). We used the Korean version of the ESS, which was validated in Korean patients. This ensured that we had successfully evaluated EDS in the participants from the Korean general population. Nevertheless, objective measurement may provide more information regarding total sleep time, sleep architecture, sleep efficacy, and other sleep derivatives. Second, we did not investigate medication use in the present study. Various medications, including anticonvulsants, antidepressants, antiemetics, antihistamines, antihypertensives, hypnotics, and muscle relaxants, may affect sleepiness (3). Third, the response rate in this study, which was 36.3%, was not high. However, we used two-stage random clustered sampling to reflect the population distribution in Korea. Moreover, there was no significant difference in the sociodemographic factors between participants in the present study and the Korean general population. Further, the prevalence of TTH and EDS was similar to that reported in previous studies. Lastly, we used 1-year time interval for the assessment of headache, TTH and without headache. Consequently, participants with TTH experienced TTH attack less than one attack per year, they were classified as participants without headache. We did not investigate participants' life-time headache or TTH and could not assess the difference in EDS prevalence between participants without TTH during their lifetimes and TTH participants experiencing less than one attack per year.

This study has several strengths. First, this study adopted the Korean version of the used questionnaires, which were previously validated for evaluating TTH, EDS, anxiety, depression, and poor sleep quality. Second, this study used a sample that reflected the Korean general population distribution, which allowed us to accurately investigate the prevalence of TTH, EDS, anxiety, depression, and poor sleep quality with a low sampling error. Third, we investigated potential covariates, including sociodemographic factors, anxiety, depression, and poor sleep quality for EDS, and analyzed the contributing factors to EDS in TTH.

## Conclusion

Although the overall prevalence of EDS in TTH individuals was not significantly different from that in individuals without headache, CTTH individuals had a higher prevalence of EDS compared to ETTH and individuals without headache. Furthermore, TTH participants with EDS experienced more severe symptoms of TTH compared to those without EDS. Therefore, EDS should be carefully investigated during the management of TTH.

## Data Availability Statement

The datasets used and/or analyzed during the current study are available from the corresponding author on reasonable request.

## Ethics Statement

The studies involving human participants were reviewed and approved by the Institutional Review Board and Ethics Committee of Hallym University Sacred Heart Hospital. The patients/participants provided their written informed consent to participate in this study.

## Author Contributions

KK: data analysis and interpretation and manuscript writing. JK and S-JC: data analysis and interpretation. W-JK, KY, and C-HY: collection and assembly of data. MC: conception and design, data analysis and interpretation, manuscript writing and critical revision.

### Conflict of Interest

The authors declare that the research was conducted in the absence of any commercial or financial relationships that could be construed as a potential conflict of interest.
